# Development of lung segmentation method in x-ray images of children based on TransResUNet

**DOI:** 10.3389/fradi.2023.1190745

**Published:** 2023-04-27

**Authors:** Lingdong Chen, Zhuo Yu, Jian Huang, Liqi Shu, Pekka Kuosmanen, Chen Shen, Xiaohui Ma, Jing Li, Chensheng Sun, Zheming Li, Ting Shu, Gang Yu

**Affiliations:** ^1^ Department of Data and Information, The Children’s Hospital Zhejiang University School of Medicine, Hangzhou, China; ^2^ Medicine Engineering and Information Research Institute for Children's Health, Sino-Finland Joint AI Laboratory for Child Health of Zhejiang Province, Hangzhou, China; ^3^ Medicine Engineering and Information Research Institute for Children's Health, National Clinical Research Center for Child Health, Hangzhou, China; ^4^ Department of Scientific Research, Huiying Medical Technology (Beijing) Co., Ltd, Beijing, China; ^5^ Department of Neurology, The Warren Alpert Medical School of Brown University, Providence, RI, United States; ^6^ Department of Scientific Research, Avaintec Oy Company, Helsinki, Finland; ^7^ Department of Radiology, Children’s Hospital, Zhejiang University School of Medicine, Hangzhou, China; ^8^ Department of Information Standardization Research,National Institute of Hospital Administration, NHC, Beijing, China; ^9^ Polytechnic Institute, Zhejiang University, Hangzhou, China

**Keywords:** children, lung segmentation, TransResUNet, chest x-ray, multi-center

## Abstract

**Background:**

Chest x-ray (CXR) is widely applied for the detection and diagnosis of children's lung diseases. Lung field segmentation in digital CXR images is a key section of many computer-aided diagnosis systems.

**Objective:**

In this study, we propose a method based on deep learning to improve the lung segmentation quality and accuracy of children's multi-center CXR images.

**Methods:**

The novelty of the proposed method is the combination of merits of TransUNet and ResUNet. The former can provide a self-attention module improving the feature learning ability of the model, while the latter can avoid the problem of network degradation.

**Results:**

Applied on the test set containing multi-center data, our model achieved a Dice score of 0.9822.

**Conclusions:**

This novel lung segmentation method proposed in this work based on TransResUNet is better than other existing medical image segmentation networks.

## 1. Introduction

With the development of modern medicine, the importance of medical imaging has become increasingly prominent. Among routine imaging examinations, x-ray examination, especially chest film examination, occupies a large proportion in children's photography due to its advantages of low dose radiation, convenient examination and low cost, accounting for more than 40% of all imaging diagnoses, of which infant chest photography accounts for more than 60% of the whole pediatric x-ray examination (1, 2). Therefore, chest x-ray has become one of the most commonly used medical diagnostic methods to detect children's lung diseases. Lung segmentation is the process of accurately identifying lung field regions and boundaries from surrounding thoracic tissue and is therefore an important first step in lung image analysis for many clinical decision support systems, such as detection and diagnosis of Covid-19 (3–5), classification of pneumonia (6), detectionof tuberculosis (7), etc. Correct identification of lung fields enables further computational analysis of these anatomical regions, such as extraction of clinically relevant features to train a machine learning algorithm for the detection of disease and abnormalities. These computational methodologies can assist physicians with making a timely, accurate medical diagnosis to improve the quality of care and outcome for patients. Lung diseases are the leading cause of death among children in many countries, mainly including bronchiolitis, bronchitis, bronchopneumonia, interstitial pneumonia, lobar pneumonia and pneumothorax. But the diagnosis of these diseases relies on the precise segmentation of the lungs in children's chest x-rays (9). Due to the immature development of the anatomical and physiological structures of preschool children, and the common thymus shadow and transverse heart in infants and young children, children's chest x-ray images generally have image problems such as the unclear boundary of lung field and severe inter-organization interference, which brings great difficulties to lung tissue segmentation (8, 9). Therefore, improving the segmentation quality and accuracy of children's lung x-ray images is of great significance to improve the diagnosis of chest x-ray film.

Despite the presence of many variations in medical image segmentation methods, these methods are broadly divided into the following five categories: medical image segmentation based on region growing arithmetic, medical image segmentation based on the deformation models, medical image segmentation based on thresholds, medical image segmentation based on graph theory, and medical image segmentation based on artificial intelligence. The traditional image segmentation method relies on artificial means to extract and select information such as edges, colors, and textures in the image. In addition to consuming considerable energy resources and workers' time, it also requires a high degree of expertise to extract useful feature information, which no longer meets the practical application requirements of medical image segmentation and recognition (10–13). With the increasing requirements for the performance of segmentation in medical imaging in recent years, Deep learning has already been extensively applied to segmentation in medical imaging. The U-Net (14) proposed in 2015 demonstrates the advantages of accurate segmentation of small targets and its scalable architecture based on encoder-decoder architecture. It achieved significant success in medical image segmentation. Many researchers have made improvements on its basis. Ummadi V (15) summarized the application of UNet and its variants in the field of medical image segmentation, including UNet++ (16), R2UNet (17), AttUNet (18), etc. In addition, Literature (19–22) also proposed or use UNet+++, ResUNet, ResUNet+, DenseResUNet, etc. for medical image segmentation. Literature et al. (23, 24) proposed 3D UNet and VNet for segmenting 3D medical images and achieved good performance. Recently, Vision transformers have achieved decent performance on computer vision tasks. Dosovitskiy et al. (25) use a pure transformer to achieve state-of-the-art performance on image segmentation data. Literature (26–29) proposed transformer-based models for the task of image segmentation. Chen et al. (27) proposed TransUNet by combining UNet and transformer for medical image segmentation. Literature (28, 29) proposed TransBTS and UNETR, which are based transformers, for 3D medical image segmentation.

In this study, we developed a novel method based on TransResUNet for the segmentation of lung areas from chest x-ray images. The TransResUNet utilized here can extract more valid information at the encoder stage and makes use of self-attention from the input feature maps. In addition, we also employ gray-scale truncation to normalize the data, and ecological operations to optimize the segmentation of the model. Experimental results on the children's x-ray dataset demonstrate the superiority of our method against other existing methods for lung segmentation.

## 2. Materials and methods

The development of the proposed model mainy includes three steps. Firstly, the image preprocessing step is to normalize the collected x-ray images. The second step is the training of the lung segmentation model based on the TransResUNet with TransUNet (27) and ResUNet (30) as the core to obtain the lung area. Then, in the image post-processing step, the segmentation performance of the model can be further improved.

### 2.1. Image preprocessing

**Figure 1:**
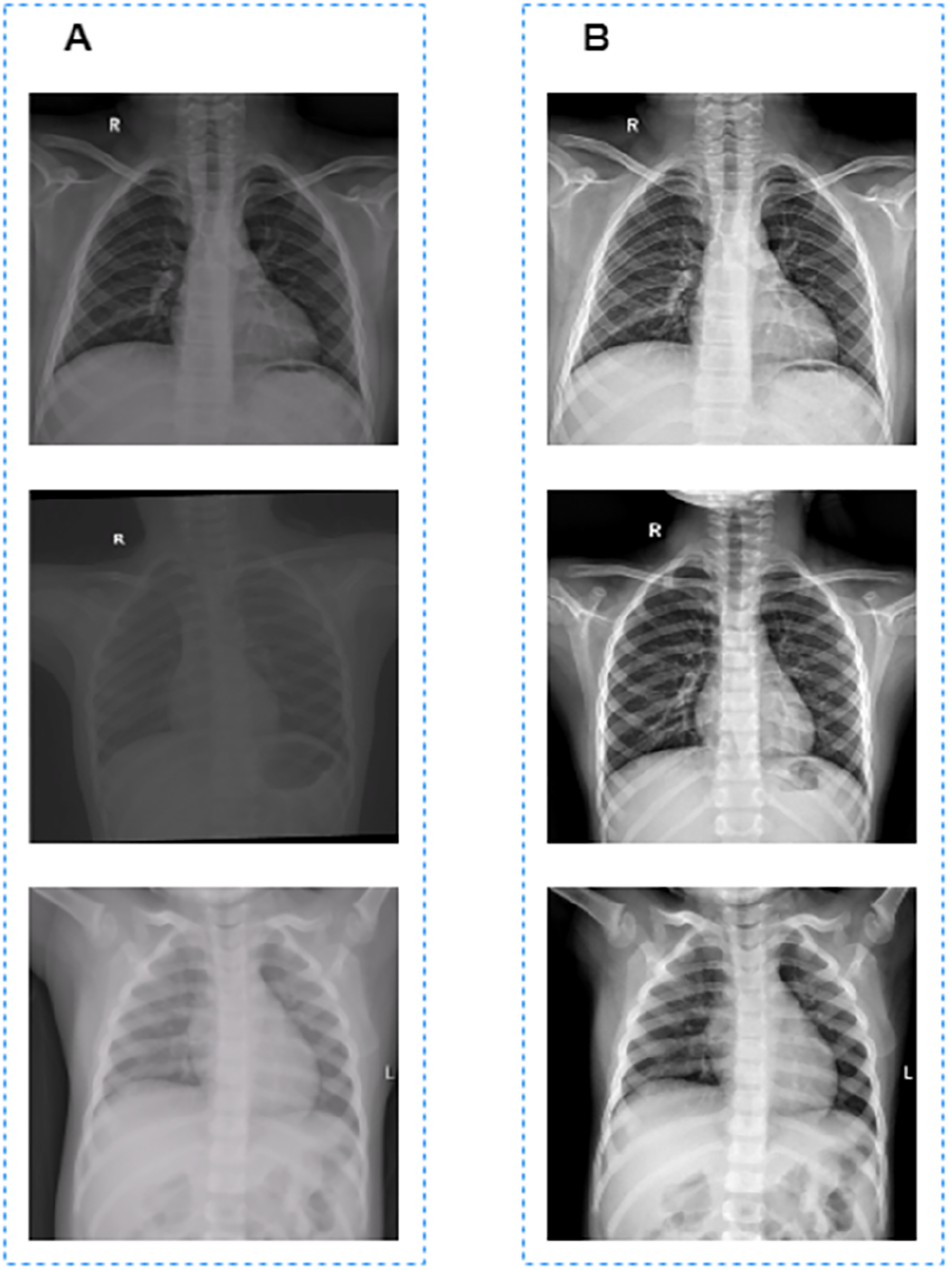
Image preprocessing results. (**A**) Images before grayscale truncation. (**B**) Images after grayscale truncation.

CXR images used to train the segmentaion model in this work were collected from different machines with different parameter settings, which resulted in the inconsistent pixel value ranges of these CXR images. If the normalization operation is used directly to these unprocessed CXR images, there will be great differences in the grayscale of the processed images, as shown in Figure 1A.

To solve this problem, we used the grayscale truncation method to preprocess the images. First, for an image with the size of x*y, we selected the 1/4 central area of the image, that is $A_{c}=\{P_{i,j}|i\in [\frac{x}{4},\frac{3x}{4}],j\in [\frac{y}{4},\frac{3y}{4}])\}$. Then the maximum and minimum values of the central area were obtained. The whole image were then grayscale truncated use [$V_{\min},V_{\max}$] with the formula as follows:

(1) $$V_{i,j}=\{\begin{array}{c} V_{\min},if\begin{array}{cc} & V_{i,j} \end{array}=<V_{\min}\\ V_{i,j},if\begin{array}{cc} & V_{\min}<V_{i,j} \end{array}<V_{\max}\\ V_{\max},if\begin{array}{cc} & V_{i,j} \end{array}>=V_{\max} \end{array},i\in [0,x],j\in [0,j]$$

The grayscale truncated image is shown in Figure 1B. Finally, the truncated images were normalized to [0, 1] for model training and validation.

### 2.2. Model traning

**Figure 2:**
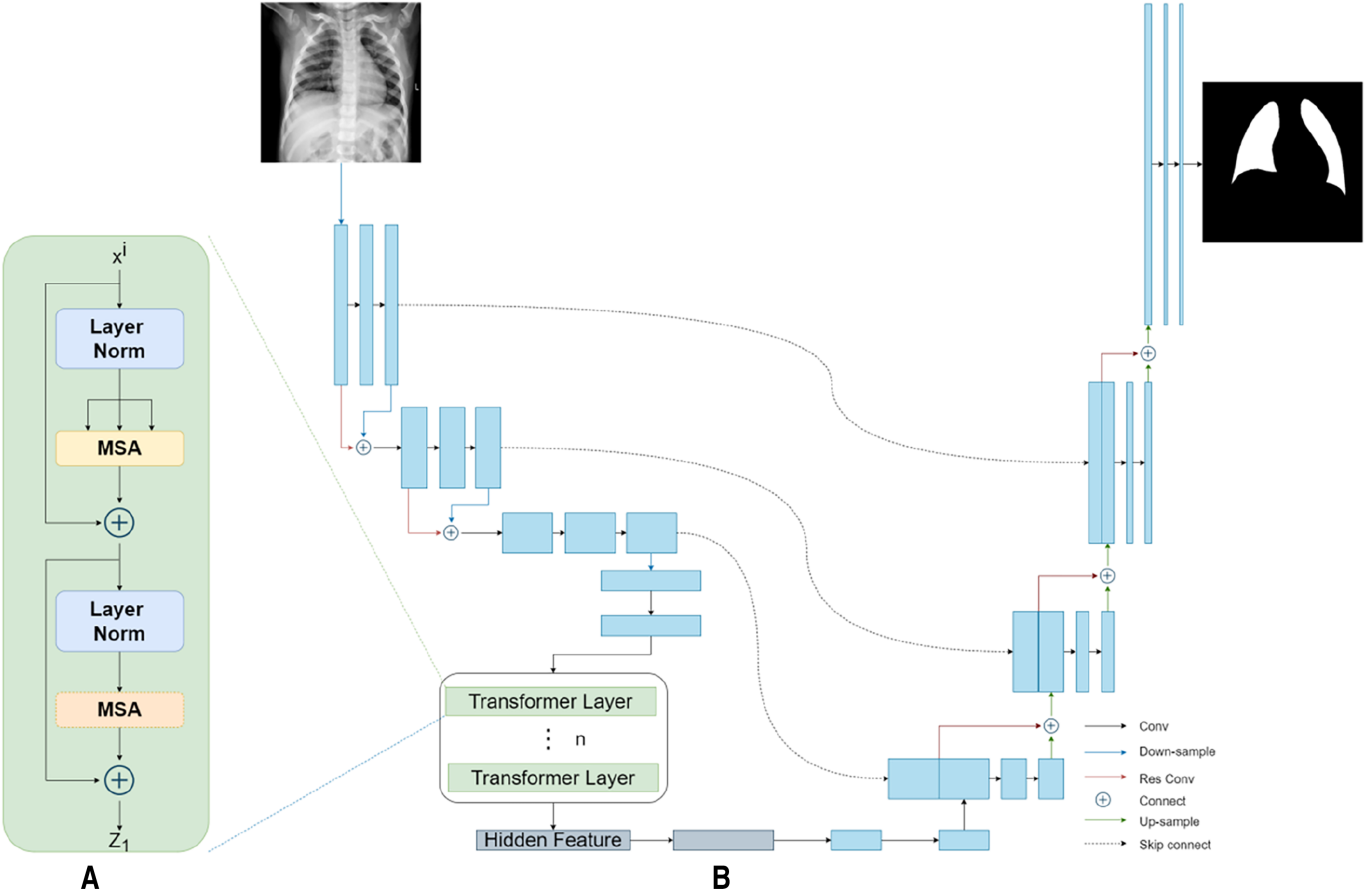
The architecture of TransResUNet. (**A**) Depicts the deconstruction schematic of Transformer Layer. (**B**) Depicts the proposed model.

Our TransResUNet model combined TransUNet and ResUNet, which were separately reported in previous works (27, 30), and further refined the combination. The original TransUNet was an encoder-decoder architecture that integrate the advantages of both Transformers and UNet. This model used CNN-Transformer as an encoder to extract global contexts and then used a UNet decoder to achieve precise localization. The TransUNet model showed satisfying performances in many medical image segmentation tasks (31, 32). The original ResUNet was a semantic segmentation neural network that combines the strengths of residual learning and UNet. This model used residual units to ease the training of deep networks. Here, we proposed a new encoder-decoder architecture called TransResUNet which equipped the classical TransUNet with residual learning unit. The model is shown in Figure 2.

#### 2.2.1. The residual learning

**Figure 3:**
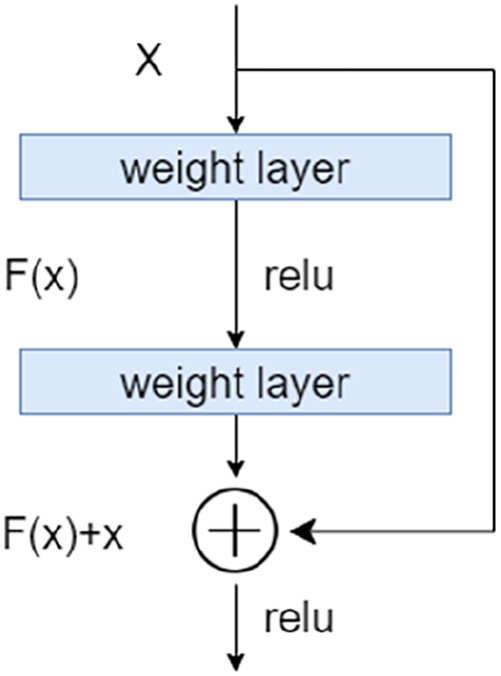
The residual learning block.

To address the degradation problem in deep networks, we used a Res learning block before each downsampling and upsampling. As shown in Figure 3, the Res learning block connected neurons in non-adjacent layers by passing the neurons of the adjacent layers, thereby weakened the strong connections between the adjacent layers.

#### 2.2.2. CNN-Transformer hybrid as encoder

Different from the common transformer model, the CNN-Transformer hybrid model was used here as the encoder. Firstly, we used the ResUNet downsampling the input image three times continuously to obtain the corresponding feature matrix $F_{1},F_{2},F_{3}$. The feature matrix were then flattened into a sequence of 2D patches according to a reported method (25). Afterwards, in order to obtain the spatial information between patches, the location information of those 2D patches were embeded. Finally, the patches with position information were inputted into the transformer encoder, which contains 12 transformer layers with each layer composing of Multihead Self-Attention (MSA) and Multi-Layer Perceptron (MLP) blocks. The output of the transformer encoder was the feature matrix $F_{t}$.

#### 2.2.3. ResUNet as decoder

In the model reported here, ResUNet was chosen as Decoder. The feature matrix $F_{t}$ and $F_{3}$ were concatenated and then unsampled twice. The feature matrix obtained from each upsampling process was concatenated with the downsampling matrix of the same size. Finally, the prediction result of each point in the input image were obtained by the segmentation model through the fully connection layer.

### 2.3. Image post-processing

**Figure 4:**
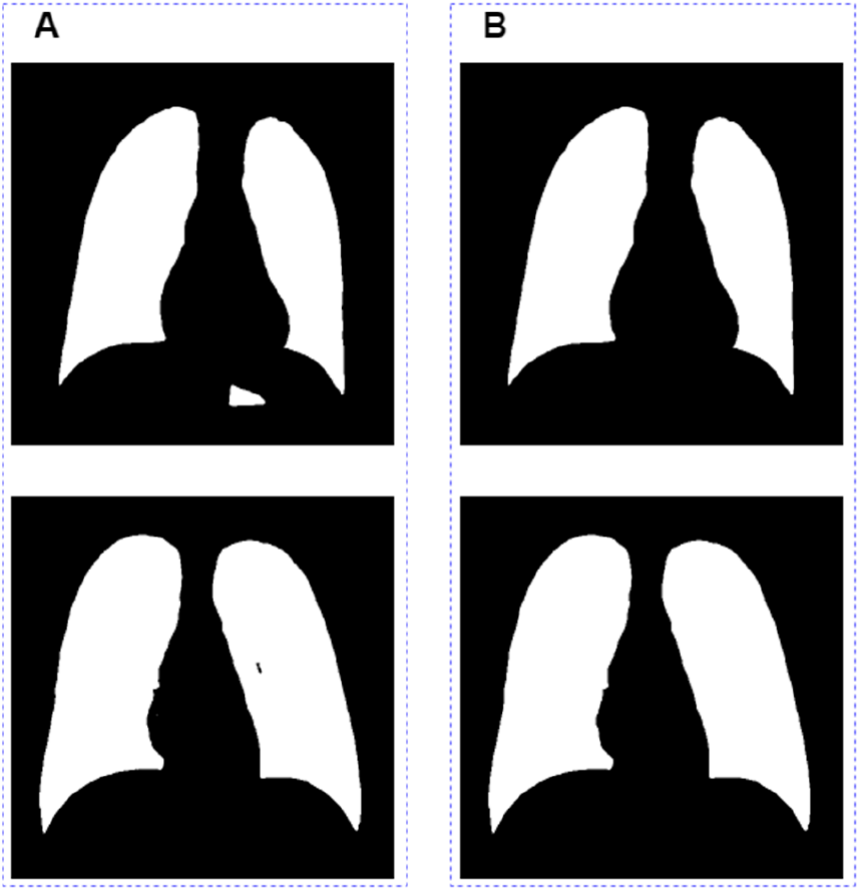
Image post-processing results. (**A**) Images before grayscale truncate. (**B**) Images after grayscale truncate.

There are two major problems in the segmentation results outputted directly from the model (Figure 4A): one is that the non-lung areas with gray levels close to that of the lung areas were identified as lungs; the other is that a small section in the lung area could not be recognized as lungs by the model. In order to further optimize the segmentation results, we removed the misidentified part by connected domain, and then used the ecological opening operation to complete the missing section of the whole lung filed. The final segamentation result after post-processing is shown in Figure 4B.

## 3. Results and discussion

### 3.1. Datasets

#### 3.1.1. X-ray scanning method

GE Definium 6000 machine was used to scan the lungs. The infants are generally photographed in a lying position. The patient is placed in a supine front-back position, with the head advanced, and the arms abducted at right angles to the body. One attendant stands on the side of the child's head and gently presses the two upper arms, the other attendant stands on the side of the child's feet and presses the abdomen and knees. The target film distance shall be 90 cm, and no filter shall be used. The older children are photographed in a standing position. The child shall be in a standing front-back position, the upper jaw is slightly raised, and the arms are abducted and placed on both sides of the body. The attendants stand on both sides of the patient, fix the child's arms and then take an x-ray. The upper end of the film shall exceed the shoulder by 2 fingers, and the target film distance shall be 180 cm. No filter shall be used. During the filming, both the patients and the attendants should take necessary protection. Before scanning, pay attention to removing metal ornaments and foreign objects to avoid artifacts.

#### 3.1.2. X-ray scanning parameters

GE Definium 6000 machine was used with the horizontal photography parameters set at 55 kV, 100 mA, 6.3 mAs, 31.5 mSec, and the vertical photography parameters set at 60 kV, 300 mA, 6.3 mAs, 31.5 mSec.

#### 3.1.3. Data set

In order to verify the performance of the proposed model, we conducted a method test on the chest x-ray image dataset of children. All data comes from the Children's Hospital of Zhejiang University School of Medicine. The children's data set includes a total of 1,158 cases. Manual segmentation was performed by two physicians with 5 years of clinical experience, and the segmentation results were used as ground truch

### 3.2. Evaluation metrics

We use the accuracy AC, sensitivity SE, specificity SP of the lung region and Dice coefficient (DI) to evaluate the accuracy of segmentation results. The first three are calculated by four variables: true positive TP, true negative TN, false positive FP, and false negative FN. The calculation formula of the Dice coefficient is as follows:

(5) $$DI(e,f)=2\frac{\left|e\cap f\right|}{\left|e|+|f\right|}$$

where *e* represents the ground truth and *f* represents the segmentation result.

### 3.3. Implementation details

We implement model in PyTorch. All training, testing and verification experiments are completed on Ubuntu 16.04 server. The basic configuration is: CPU Intel E5-1650 3.50 GHz, 64G DDR4 memory, the graphics card is RTX 2080Ti. All annotation work is done in RadCloud (Huiying Medical Technology (Beijing) Co., Ltd). All models were trained with the batch size of 2, using the RMSprop optimizer (33) with an initial learning rate of 0.001 for 20 epochs. All images were reshaped to 512*512. We use R50-ViT-B_16 (25) architecture with *L* = 12 layers and an embedding size of *K* = 768. We split the data into train, validation and test with a ratio of 70:10:20, and their numbers are 810, 116, and 232, respectively.

### 3.4. Quantitative evaluations

We selected UNet (14), UNet++ (16), UNet+++ (19), AttUNet (18), ResUNet (32), TransUNet (27), and TransResUNet for training and testing on the dataset. As shown in Table 1, TransResUNet outperforms the state-of-the-art methods in our test datasets. The overall average Dice score of TransResUNet is 98.02% which outperforms the second by 1.06%. The overall average AC, SE and SP of all models are very close because the lung area has a certain grayscale difference from the surrounding area. Nonetheless, TransResUNet achieves the highest scores on both SE and SP, indicating that it performs well for the lung segmentation task.

**Table 1:** Quantitative comparisons of the lung segmentation performance in test dataset.

Method	AC	SE	SP	Dice
UNet	98.82%	96.68%	99.31%	0.9694
UNet++	98.81%	96.64%	99.30%	0.9692
UNet+++	98.89%	96.95%	99.33%	0.9709
AttUNet	**98** **.** **90%**	97.03%	99.33%	0.9716
ResUNet	98.84%	96.67%	99.33%	0.9698
TransUNet	98.85%	96.73%	99.33%	0.9701
TransResUNet	98.88%	**97** **.** **92%**	**99** **.** **44%**	**0.9822**

The bold values represent the highest value of each evaluation indicator. They are used to show that our model achieves the best performance in most indicators.

### 3.5. Qualitative results

**Figure 5:**
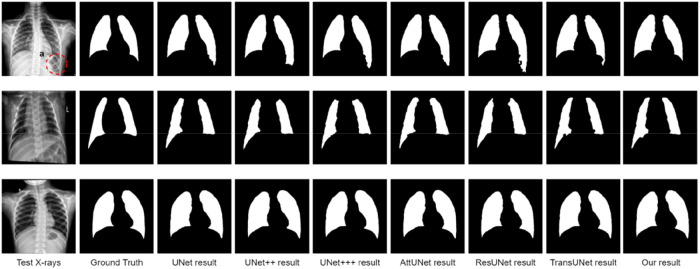
Qualitative comparison of different models in the test dataset.

Qualitative lung segmentation comparisons are presented in Figure 5. TransResUNet shows improved lung segmentation performance. In Figure 5, the first row and second row demonstrate a clear segmentation against surrounding tissues. For example, the area (Figure 5A) below the lungs in the first row indicates that TransResUNet has sufficient learning for global information. For areas with clear lung edges, such as the third rows in Figure 5, the segmentation performance of each model is relatively close. However, like other models, our model is not smooth at the edges because the edges of the lungs are occluded by the bright ribs, but this does not reflect the segmentation performance of the main lung regions.

## 4. Conclusion

In this work, a novel lung segmentation method based on TransResUNet in chest x-ray images has been reported. First of all, we truncated the raw x-ray images with the central region gray value range of the images to reduce the inconsistence caused by different machine acquisitions settings. When developing the segmentation model, we used the encoder part of ResUNet to replace the encoder part of classical TransUNet, thereby improving the feature extraction ability of the model in the downsampling stage. Finally, ecological operations were used to further optimize the results of segmentation. The experimental results demonstrated that the methods of this paper outperform the original TransUNet model and other reviously reported models. In the future, we will consider eliminating the rib region before segmentation, so as to improve the segmentation performance of the model, so that the model can be better applied to clinical diagnosis and research on other lung diseases.

## Data Availability

The original contributions presented in the study are included in the article, further inquiries can be directed to the corresponding authors.
